# A technique for setting analytical thresholds in massively parallel sequencing-based forensic DNA analysis

**DOI:** 10.1371/journal.pone.0178005

**Published:** 2017-05-18

**Authors:** Brian Young, Jonathan L. King, Bruce Budowle, Luigi Armogida

**Affiliations:** 1 NicheVision Forensics, Akron, Ohio, United States of America; 2 Center for Human Identification, University of North Texas Health Science Center, 3500 Camp Bowie Blvd., Fort Worth, TX, United States of America; 3 Center of Excellence in Genomic Medicine Research (CEGMR), King Abdulaziz University, Jeddah, Saudi Arabia; University of Helsinki, FINLAND

## Abstract

Amplicon (targeted) sequencing by massively parallel sequencing (PCR-MPS) is a potential method for use in forensic DNA analyses. In this application, PCR-MPS may supplement or replace other instrumental analysis methods such as capillary electrophoresis and Sanger sequencing for STR and mitochondrial DNA typing, respectively. PCR-MPS also may enable the expansion of forensic DNA analysis methods to include new marker systems such as single nucleotide polymorphisms (SNPs) and insertion/deletions (indels) that currently are assayable using various instrumental analysis methods including microarray and quantitative PCR. Acceptance of PCR-MPS as a forensic method will depend in part upon developing protocols and criteria that define the limitations of a method, including a defensible analytical threshold or method detection limit. This paper describes an approach to establish objective analytical thresholds suitable for multiplexed PCR-MPS methods. A definition is proposed for PCR-MPS method background noise, and an analytical threshold based on background noise is described.

## Introduction

Since its inception, massively parallel sequencing (MPS) technology has had enormous impact on genomic characterization. Now, MPS technology is beginning to be applied to forensic DNA analyses. Sequencer instrumentation and forensic kit manufacturers are developing hardware, software, PCR primers and reagents necessary to support implementation of PCR-MPS systems as routine methods in forensic laboratories [1–11]. While currently considered as an adjunct or complementary system, PCR-MPS could supplement or possibly replace existing forensic analysis methods such as capillary electrophoresis (CE)-based DNA fragment analysis of short tandem repeat (STR) loci and Sanger sequencing of mitochondrial DNA (mtDNA) hypervariable regions. In addition, PCR-MPS can analyze substantially more genetic markers of forensic interest including novel STRs, single nucleotide polymorphisms (SNPs), Alu elements, insertions/deletions (indels) and microhaplotypes. The high throughput capability of PCR-MPS, which enables analysis of a wide range of forensic markers and samples in a single assay, is one of the attractive features of this technology.

One challenge to implementing PCR-MPS as a routine forensic DNA analysis method is defining an analytical threshold (AT) in a way suitable to support meaningful interpretation of DNA evidence results. The AT is critically important to allele detection, and a properly set AT provides a limit above which all method responses shall be interpreted. Conversely, the AT serves to prevent over-interpretation of data. This latter aspect is especially important in PCR-MPS methods where it is possible to examine the read sequences of clones of individual amplicon molecules from the PCR.

Assignment of ATs in instrumental analysis often is approached by measuring method background noise in the absence of signal and then using features of the noise data to assign an AT. Two commonly used features of background noise in this application are the range and distribution [12] of the noise response intensities. False positive rates can be controlled by setting ATs sufficiently outside the range of or sufficiently far into the tail of a distribution fitted to the observed background noise. However, there currently is no agreed upon definition of background noise in PCR-MPS data in the field of forensic DNA analysis. Consequently, to the authors’ knowledge, there are no established methods available for setting PCR-MPS forensic method ATs based on background noise. In contrast, there has been some agreement that sequence read coverage provides a useful definition for signal, and several investigators have defined AT values based on signal intensity [2,3,13,14]. This approach has been adopted by commercial vendors of forensic DNA analysis kits [15,16]. However, this approach is not based on an underpinning concept of method noise and does not facilitate calculation of error rates (i.e., false positive rates) which are critically important in forensic DNA analysis. Here one possible technique is described for setting ATs in PCR-MPS methods using background noise.

## Materials and methods

### Sample preparation

A total of four whole blood DNA samples were collected via venipuncture, processed, and data maintained in accordance with the policies and procedures approved by the Office for the Protection of Human Subjects Institutional Review Board for the University of North Texas Health Science Center in Fort Worth, TX (#2010–132). DNA was extracted using the QIAamp DNA Blood Mini Kit (Qiagen; Hilden, Germany) according to manufacturer’s recommendations. DNA quantities were determined using the Qubit™ dsDNA HS Quantification Kit and Qubit™ 2.0 Flurometer (Thermo Fisher, San Francisco, CA, USA). Samples were normalized to 0.2 ng/μL prior to MPS.

### Sequencing

Library preparation and sequencing was performed as described by Novroski et al. [12]. Briefly, genomic targets were amplified and tagged using ForenSeq oligonucleotide primer mix B and a GeneAmp1 PCR System 9700 thermal cycler following the manufacturer’s recommended beta protocol. Multiplexing index sequences and adapters for cluster generation were added via enrichment amplification using a GeneAmp1 PCR System 9700 thermal cycler following the manufacturer's protocol. Libraries were normalized and pooled for sequencing in batches of 32 samples using 10 μL volumes. The ForenSeq primer set B includes 27 autosomal STR loci, 94 identity informative SNP loci, 22 phenotype informative SNP loci, 56 ancestry informative SNP loci, 25 Y STR markers and 7 X STR markers. All loci were sequenced, but only the autosomal STR marker data are reported here.

Sequencing was performed on the MiSeq FGx Forensic Genomics System (351 x 31 bp) (Illumina) according to the manufacturer’s protocol.

### Data analysis

The FASTQ files produced by the Illumina ForenSeq™ pipeline were transferred from the sequencing workstation and analyzed using STRait Razor^TM^ software [2] instead of the ForenSeq^TM^ Universal Analysis software. Briefly, reads corresponding to autosomal STR loci were identified and binned via matching to primer binding site sequences using the highest stringency setting (zero mis-matches allowed). Read sub-sequences corresponding to the STR variable regions were isolated via high-stringency matching to 10-nucleotide flanking sequences by entering the flanking sequences into the STRait Razor configuration file. Within each sample and each locus, read sub-sequences were binned by unique sequence into sets of mutually exclusive and exhaustive unique-sequence bins. The unique sub-sequence of each bin and the count of reads containing that unique sequence were formatted for export to Excel using a custom Python script. Sub-sequences were categorized by visual inspection into three categories: signal corresponding to the known genotype of the samples, stutter artifacts, and background noise.

### Definition of background noise

In its broadest sense, background noise is method response in the absence of analyte signal, and while with PCR-CE some individuals have recommended that an AT be set based on negative controls [17], positive controls are better suited for establishing an AT as noise is not contributed solely from the instrument (i.e., there is PCR noise to consider as well) [12,18]. The selection of a negative control for establishing an AT for PCR-MPS data is not justified as the instrument itself does not generate noise that can be confused with signal. The signal response in PCR-MPS methods consists of read sequences. Under optimal conditions, negative controls should be devoid of read sequences. Background noise in positive controls can be isolated numerous ways [12] including filtering out responses from alleles and molecular artifacts [11,19]. Here, we define background noise as PCR-MPS method response in positive controls that is not analyte signal and not molecular artifact. This definition is contingent upon a precise definition of analyte signal and molecular artifact. Here we adopt the definition for analyte signal used by others as the count intensity of sequence reads that cover a targeted genetic locus [1–11]. These read sub-sequences match the sequence of one or both authentic alleles at a locus. Critical to our method is a precise description of the genetic locus for which coverage is measured. STR loci targeted in forensic DNA analysis are polymorphic often with unknown numbers of undiscovered rare types making it impractical to describe the total variation of a locus. Therefore, loci under analysis are defined and delimited by short sub-sequences in stable regions flanking the genomic region of interest. The term “flanking sequence landmarks” (FSL) is applied to these sub-sequences. PCR primers define the DNA fragments available for forensic analysis. However, DNA sequences of these fragments are difficult to read, and often less than the entire fragment is polymorphic. The FSL concept allows delineation of PCR fragment subsequences for use in forensic analysis. The FSL-defined loci may consist of the entire PCR-amplified DNA fragment, or any sub-segment of that fragment. Here we demonstrate the method by focusing only on the variable STR regions of PCR fragments [2,3]. Read sequences identifiable as the result of systematic method error are molecular artifacts. The most important molecular artifact in forensic DNA analysis of STR loci is the stutter artifact [20]. All three categories of sub-sequence types for a given locus (allele, artifact, noise) arise from measurement of the same flanking sequence landmarks (FSL)-proscribed DNA sub-segment. Filtering for DNA segments containing syntenic pairs of FSLs makes AT calculations specific to loci and makes the method robust to the number and type of other loci or samples that may be multiplexed in a PCR-MPS workflow. Reads not associated with specific loci such as those arising from primer-dimers or PCR hybrids are eliminated from the locus-centric type frequency spectra.

**Fig 1:**
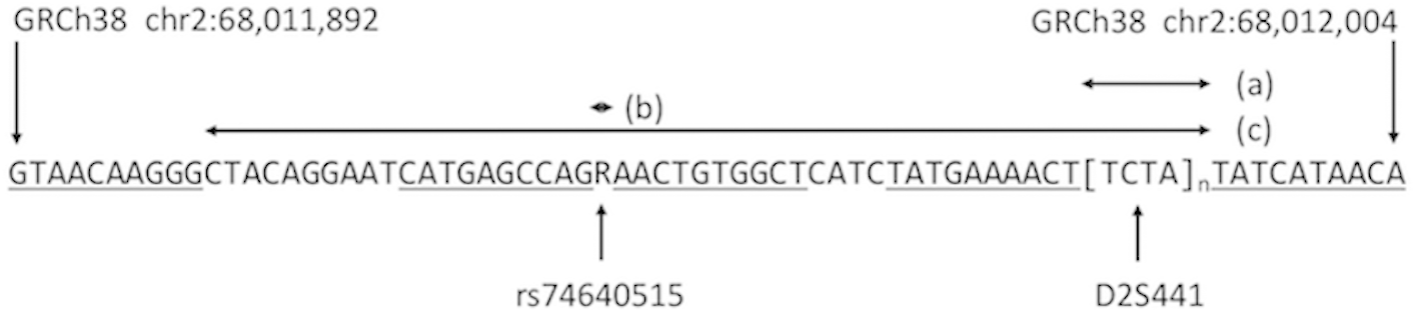
Illustration of locus definitions as defined by flanking sequence landmarks (FSLs). The genomic sub-sequence between GRCh38/hg38 positions chr2:68,011,892 and chr2:68,012,004 includes the forensic STR locus D2S441 and SNP locus rs74640515. Flanking sub-sequences can be used as landmarks to delimit and define sub-segments of PCR amplicons for forensic analysis. FSLs of 10 nucleotides are indicated in underlined font. FSLs need not be unique in the genome, but only unique within the extent of the genome sequenced in an assay. Three separate locus definitions are illustrated: (a) the polymorphic repeat region of the STR locus D2S441, (b) The polymorphic SNP locus rs74640515, and (c) the haplotype sub-segment including both D2S441 and rs74640515. FSLs may be set equal to the PCR binding sites, making the delimited locus the entire PCR amplicon.

The current practice in forensic DNA analysis is to amplify by PCR DNA fragments that include repeat region of interest and flanking regions defined by the primers used. For example, the forensic STR locus D2S441 is comprised of a tandemly-repeated TCTA tetramer that is known to vary from approximately 8 to 17 repeats (32 to 68 nucleotides) in human populations [21], and the PCR primers targeting this locus are located outside this variable region to produce amplicons compatible with size windows used in library preparation. In current forensic practice these fragment size windows are approximately 200 to 400 nucleotides [15,16]. Thus, the genetic locus for which coverage is measured can comprise the entire amplified segment, only the D2S441 variable region proper, or some other sub-segment of the amplicon. Several investigators have pointed out the forensic utility of considering more than one polymorphic locus within amplicons [22–24], i.e., treating the amplicon region as a haplotype. Using FSL to define the locus under analysis, the technique described herein is robust to potentially differing forensic definitions (or targets) of an amplified locus. The flexibility of FSL-defined loci is illustrated in Fig 1 where three possible locus definitions are presented for a single DNA sub-segment amplified in the Illumina ForenSeq^TM^ primer set panel. The locus under analysis may be any length ranging from one nucleotide to many nucleotides up to the entire length of sequence reads. FSL precisely define PCR amplicon sub- segments and represent an alternative to sub-segment definitions based on coordinates in a genomic assembly. While genomic coordinates must be separately specified for each assembly, FSL-based definitions do not change with assembly. The entire set of FSL used to analyze each of the 26 STR loci examined in this study is presented in S1 Table.

### Molecular artifacts

Molecular artifacts that may be confused with authentic alleles require special attention in forensic DNA analyses. In PCR-CE methods focused on the analysis of STR loci, the main detectable molecular artifact is stutter that is one repeat motif shorter, and less often two repeats shorter or one repeat motif longer than authentic alleles. However, the nature of MPS data allows observation of molecular artifacts not normally detectable in PCR-CE data. Stutter artifacts that differ from alleles by two or more motif repeats are commonly observed in PCR-MPS data, as well as complex stutter products where different repeat motifs in the same STR locus stutter separately. Stutter artifacts can be identified as fragments, or sub-segments of fragments, whose length places them in “stutter position” of other sequences present in the sample [11]. In rare situations, the increase in DNA length at one motif may cancel out a decrease in DNA length at a second motif in the same STR locus. This molecular artifact would not be “in stutter position” or noticed in PCR-CE analysis, but is considered a molecular artifact in PCR-MPS data. Hence, we define stutter artifacts in PCR-MPS data as sequences that differ from authentic allelic sequences solely by increases or decreases in repeat number at one or more repeat motifs and which exhibit count intensities within the range expected. This definition accommodates stutter artifacts that may be the same length as the parent allele, as well as stutter artifacts from any length repeat motif including homopolymers stretches. Singletons (i.e., sequences observed only once in the data set) are considered background noise regardless of sequence in this study.

In sequence-defined alleles, a tie-breaker convention is needed when categorizing ambiguous sequences that can be interpreted as arising either from a PCR strand slippage error or as a random base substitution error. This situation arises in compound-repeat loci where adjacent repeat motifs differ by a single nucleotide, and where at least one of the motifs is repeated only once or twice. For example, across the four samples, locus D3S1358 exhibits compound-repeat allele sequences of the form [TCTA]_1_[TCTG]_1–2_[TCTA]_13–15_. Minor simple-repeat non-allele sequences of the form [TCTA]_13–17_ are present in all samples due to loss of the [TCTG]n motif. Discounting contamination, the observed minor sequences can arise from one or more G→A base substitution errors, one or more strand-slippage errors (eliminating the [TCTG] motif), or combinations of base substitutions and strand-slippage errors. The relative likelihood of these alternatives is presently unknown; therefore, a heuristic rule is needed. Where a sequence could not be assigned definitively as stutter, then the ambiguous sequence was categorized as background noise. A total of 14 ambiguous sequences types of this sort, representing just 60 reads out of a total of 199,442 reads, were observed at 5 loci (D3S1358, D8S1179, D9S1122, vWA and D19S482). More sophisticated heuristics may be possible in the future as rates and patterns of motif-specific stutter and sequence-specific base substitution become better characterized.

Molecular artifacts also can arise from base substitutions, insertions or deletions that occur in a sequence-specific manner. Sequence-specific error patterns differ by sequencing platform, where Illumina sequencer platforms predominantly generate base substitution errors [25], and ThermoFisher (or Ion Torrent) sequencer platforms generate insertion-deletion errors around nucleotide homopolymers [26]. Base substitution and insertion/deletion errors clearly distinguishable from background noise have not been reported at any substantial level for Illumina reversible-terminator sequencing, and therefore we do not consider them here. All allelic, artefactual and background noise sequences for all loci in sample numbers T9046, T23499, T28788 and T36814 are presented in S2, S3, S4 and S5 Tables respectively.

### Calculation of analytical thresholds

ATs were calculated based on PCR-MPS method background noise responses as recommended by the FBI Scientific Working Group on DNA Analysis Methods (SWGDAM) [17], using a simple method that is easily implemented in practice. The range of background noise intensities for a locus is calculated by subtracting the minimum observed noise intensity from the maximum observed noise intensity. The locus-specific range of noise intensities is multiplied by a scaling constant (***c***) to yield an AT (Eq 1). The magnitude of the scaling constant can be varied to balance the level of protection against non-reproducible noise peaks exceeding the AT against the risk of allelic data loss. Increasing ***c*** increases protection against noise peaks exceeding the AT and decreasing c increases the risk of allelic loss. The scaling factor appropriate for a given laboratory and method can be operationally derived from empirical data using STRait Razor with a minimum threshold of zero to reveal all background noise.

$$AT=c\times \left(Noise_{max}-Noise_{min}\right)$$ (1)

Here, stutter artifacts are identified by their characteristic nucleotide sequences, a procedure that permits comprehensive isolation of artifacts. The application of Eq 1 to background noise absent all alleles and all stutter artifacts identifiable by sequence is called Method A. In mainstream PCR-CE forensic methods, stutter artifacts are commonly limited to fragments exhibiting lengths corresponding to N-1 where N represents the number of tandem repeats. For purposes of illustrating the impact of this alternative definition of stutter artifact, sequence data were manually curated to isolate only N-1 stutter. All remaining stutter artifacts including N-2 and N+1 artifacts remained in background noise. The application of Eq 1 to background noise absent all alleles and only N-1 stutter artifacts is called Method B.

## Results and discussion

When PCR-MPS read sub-sequences are binned by unique sequence, then a mutually exclusive and exhaustive set of sub-sequence types can be elucidated. Together, the sub-sequence types (hereafter called types) exhibit a frequency spectrum that includes a few relatively abundant types corresponding to authentic alleles, along with many low-abundance types corresponding to background noise. Between these two extremes are types that correspond to molecular artifacts with abundances between allele types and noise types. A total of 199,442 read sub-sequences (hereafter called tokens) was observed across all four samples. These tokens were categorized into 190 allele types, 485 stutter artifact types, and 7,134 noise types. Most tokens (87%) correspond to authentic alleles, and most types (91%) correspond to noise. In single-source samples, the allele sequences are significantly more abundant than other types, and the frequency spectrum of allele types does not overlap with the frequency spectrums for molecular artifact or noise types. In contrast, the frequency spectrums of artifact and noise types can overlap. Example type frequency spectrums for simple and compound loci are presented in Table 1. Type frequency spectrums for all loci and samples are available in S2–S5 Tables.

**Table 1:** Frequency spectrum of the exhaustive and mutually exclusive set of sequence types generated for simple and compound repeat loci D5S818 and D12S391, respectively, observed in sample T36814. The number of individual reads (tokens) comprising each type is indicated by N, and counts of types one repeat motif shorter than either allele are highlighted bold font. For ease of reading, selected repeat motifs are bracketed with a number following the bracket indicating the number of tandem repeats.

Type Category	D5S818		D12S391	
	N	Sequence	N	Sequence
Allele	381	[AGAT]12	542	[AGAT]12[AGAC]6AGAT
	294	[AGAT]11	377	[AGAT]13[AGAC]6AGAT
Molecular Artifact	9	[AGAT]13	**84**	**[AGAT]11[AGAC]6AGAT**
	**7**	**[AGAT]10**	**19**	**[AGAT]12[AGAC]5AGAT**
	2	[AGAT]9	**13**	**[AGAT]13[AGAC]5AGAT**
			9	[AGAT]14[AGAC]5AGAT
			6	[AGAT]10[AGAC]6AGAT
			3	[AGAT]12[AGAC]7AGAT
			3	[AGAT]11[AGAC]5AGAT
			3	[AGAT]11[AGAC]7AGAT
			3	AGGT[AGAT]11[AGAC]6AGAT
Background Noise	2	[AGAT]2TGAT[AGAT]9	2	AGTT[AGAT]11[AGAC]6AGAT
	2	[AGAT]8AGAC[AGAT]3	2	[AGAT]10GGATAGAT[AGAC]6AGAT
	1	TGAT[AGAT]11	1	AGATGGAT[AGAT]11[AGAC]6AGAT
	1	TGAT[AGAT]10	1	AGATAGGT[AGAT]12[AGAC]6AGAT
	1	TGAT[AGAT]9	1	AGATAGGT[AGAT]10[AGAC]6AGAT
	1	AGTT[AGAT]11	1	AGATAGCT[AGAT]8AGCTAGAT[AGAC]6AGAT
	1	AGCT[AGAT]11	1	[AGAT]2AGCT[AGAT]10[AGAC]6AGAT
	1	AGATAGCT[AGAT]4AGAA[AGAT]5	1	[AGAT]3CGAT[AGAT]9[AGAC]6AGAT
	1	[AGAT]2GGAT[AGAT]9	1	[AGAT]3AGGT[AGAT]8[AGAC]6AGAT
	1	[AGAT]2CGAT[AGAT]9	1	[AGAT]3AGCT[AGAT]8[AGAC]6AGAT
	1	[AGAT]2AGCT[AGAT]9	1	[AGAT]5CGAT[AGAT]6[AGAC]6AGAT
	1	[AGAT]3TGAT[AGAT]8	1	[AGAT]5AGTT[AGAT]5[AGAC]6AGAT
	1	[AGAT]3CGAT[AGAT]8	1	[AGAT]7AGTTAGATAGCT[AGAT]3[AGAC]6AGAT
	1	[AGAT]3AGTT[AGAT]8	1	[AGAT]10TGATAGAT[AGAC]6AGAT
	1	[AGAT]3AGGT[AGAT]7	1	[AGAT]11ATAT[AGAC]6AGAT
	1	[AGAT]4AGGT[AGAT]7	1	[AGAT]12GGAT[AGAC]6AGAT
	1	[AGAT]4AGCTAGATAGTTTGTT[AGAT]3	1	[AGAT]13[AGAC]5AAACAGAT
	1	[AGAT]6CGAT[AGAT]4	1	[AGAT]12[AGAC]2TGAC[AGAC]3AGAT
	1	[AGAT]7AGCTAGCT[AGAT]3	1	[AGAT]12[AGAC]4AGATAGACAGAT
	1	[AGAT]8GGAT[AGAT]3	1	[AGAT]12[AGAC]4AGAT
	1	[AGAT]9GGAT[AGAT]2	1	[AGAT]12[AGAC]6AGGT
	1	[AGAT]10CGATAGAT	1	[AGAT]12[AGAC]7
	1	[AGAT]11CGAT	1	[AGAT]11AGAA[AGAC]6AGAT
	1	[AGAT]12AGAC	1	[AGAT]9[AGAC]6AGAT
	1	[AGAT]8AAAT[AGAT]2	1	[AGAT]8AGAC[AGAT]3[AGAC]6AGAT
	1	[AGAT]7AGAA[AGAT]4	1	[AGAT]8AGAA[AGAT]3[AGAC]6AGAT
	1	AGATAGAC[AGAT]10	1	[AGAT]6AGAG[AGAT]6[AGAC]6AGAT
	1	AGAC[AGAT]11	1	[AGAT]6AGAG[AGAT]4[AGAC]6AGAT
	1	ACAT[AGAT]2AGCT[AGAT]7	1	[AGAT]5AGAC[AGAT]5[AGAC]6AGAT
			1	[AGAT]3AGAC[AGAT]7[AGAC]6AGAT
			1	[AGAT]2AAAT[AGAT]10[AGAC]6AGAT
			1	AGATAGAA[AGAT]10[AGAC]6AGAT
			1	AGAG[AGAT]11[AGAC]6AGAT

The three categories of sequence types described here are common to all PCR-MPS methods and have been reported previously [e.g. 9,10,11]. The allele category represents correctly sequenced DNA template and any sequencing method with reasonable fidelity will produce sequence types of this category. Stutter artifacts are a product of PCR amplification of DNA template containing STRs. Stutter artifacts are created prior to downstream sequencing and therefore are universally present in STR analysis methods that include prior amplification by PCR. When binned by length rather than sequence, stutter artifacts observed in PCR-MPS methods exhibit intensity patterns similar to those observed in PCR-CE methods. As in PCR-CE, stutter intensities differ by locus and the read count intensities of N-1 stutter products are roughly 10% of the read count intensity of parent alleles, and read count intensities of N-2 stutter products are roughly the square of the percentage intensities of N-1 stutter. The background noise category represents all sequences that can be associated with a locus but which cannot be categorized as either allele or stutter. Sequence types of this category can arise from numerous sources including base substitution error, and can occur either in the PCR stage or in the sequencing stage. All sequencing platforms generate base substitution error at characteristic rates, and thus background noise is always present in these methods. Thus, while all PCR-MPS methods can be expected to produce sequence types of all three categories (allele, stutter and background noise), the relative intensities of sequence types in these categories will be method specific. One difference between PCR-CE and PCR-MPS methods is that the latter methods are known to produce sequence-specific error (SSE) characteristic of the MPS platform. Whenever it is present, SSE will fall to background noise in the sequence type categorization described here. When present, SSE will elevate background noise and consequently elevate AT calculated based on background noise. Future methods might reliably identify SSE in forensic PCR-MPS methods, allowing these sequence types to be categorized as molecular artifacts thereby lowering background noise levels.

### AT values

**Fig 2:**
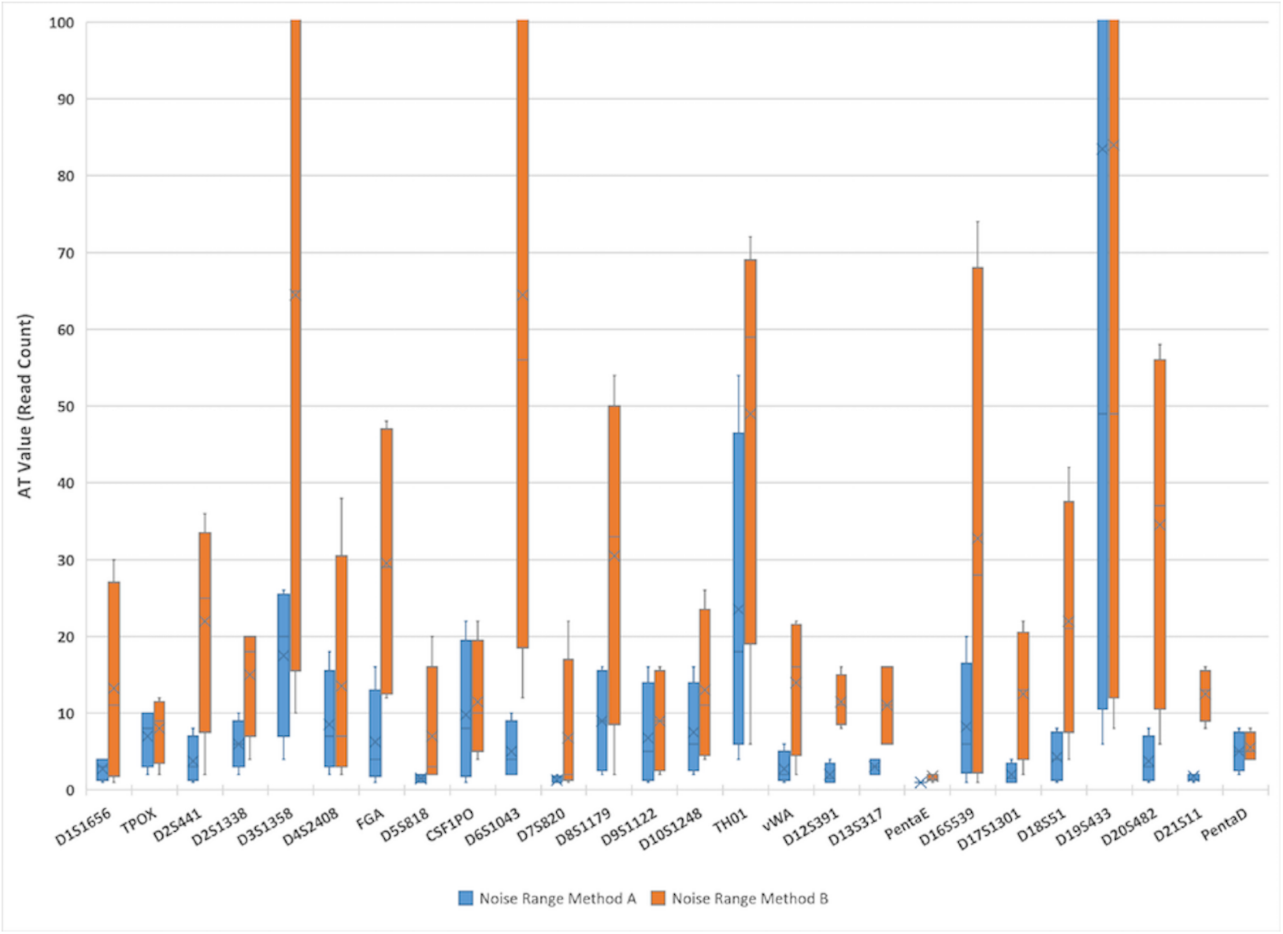
Per-locus analytical threshold (AT) values calculated from the range of observed noise by each of two methods. AT values by method A (blue boxes) are set at twice the range of observed per-locus noise intensities, where per-locus noise is defined as responses not attributable to alleles or molecular artifacts (stutter). AT values by method B (orange boxes) are set using the same formula, but where per-locus noise is defined as responses not attributable to authentic alleles or N-1 molecular artifacts. Boxes summarize data across 4 DNA samples, and the plot Y-axis is truncated at 100 read counts for purposes of readability.

AT values were calculated using background noise data by Eq 1 and compared to the default AT calculation method used for the Illumina ForenSeq^TM^ Universal Analysis Software [15]. All loci within all samples exhibited background noise, meaning that the minimum noise intensity at all loci was at least 1 read. Maximum intensities for background noise vary by sample, and by locus within sample. For example, the maximum intensity across all samples and all loci was 116 reads at locus D19S433 in sample number T9046; whereas the maximum intensity for across all loci within sample number T28788 was only 4 reads also at the D19S433 locus. Background noise intensities for all samples and all loci within samples are presented in S2–S5 Tables. Given a maximum experiment-wide background noise intensity of 116, then by applying Eq 1 with a scaling factor c = 2, an experiment-wide AT of 230 read counts is obtained. The relatively high observed experiment-wide variability makes the experiment-wide AT level overly conservative for some loci. Here 25 of 190 authentic alleles fell below the calculated experiment-wide AT of 230 read counts, indicating that experiment-wide AT may be less appropriate for PCR-MPS methods as compared to PCR-CE methods. Excluding the loci TH01 and D19S433, per-locus AT values ranged from 1 to 24 read counts for loci in this data set. These two loci exhibited higher background noise and were the determinants of experiment- and sample-wide AT levels. In current forensic PCR-CE methods observed molecular artifacts are often limited to DNA fragment lengths that size to N-1 (and to a much lesser extent N+1). For comparison purposes ATs were recalculated using baseline noise re-expressed as method responses excluding authentic alleles and only stutter of length N-1. AT calculated by this method (i.e., method B) were generally higher than AT values calculated when all molecular artifacts were removed (i.e., method A) (Fig 2). In the case of method B, the maximum per-locus counts at 80 of 104 loci were due to N+1 or N-2 stutter artifacts. This definition, when applied to PCR-MPS data, appears conservative and wasteful of information because molecular artifacts other than N-1 stutter are clearly observable in PCR-MPS data. As noted, AT values are calculated based on background noise. Therefore, the method is robust to the level of signal present, whether read counts of stutter artifacts are added back to the read counts of their putative parent alleles, or whether data are normalized or re-scaled. An additional benefit of this approach is that AT and allele (signal) intensities are expressed in the same measurement unit. Here, both AT and allele intensities are expressed in read counts. AT values are a function of noise intensities meaning that comparing AT values to allele (i.e. signal) intensities becomes a proper comparison of signal and noise.

### Comparison to 1.5% of locus coverage

**Fig 3:**
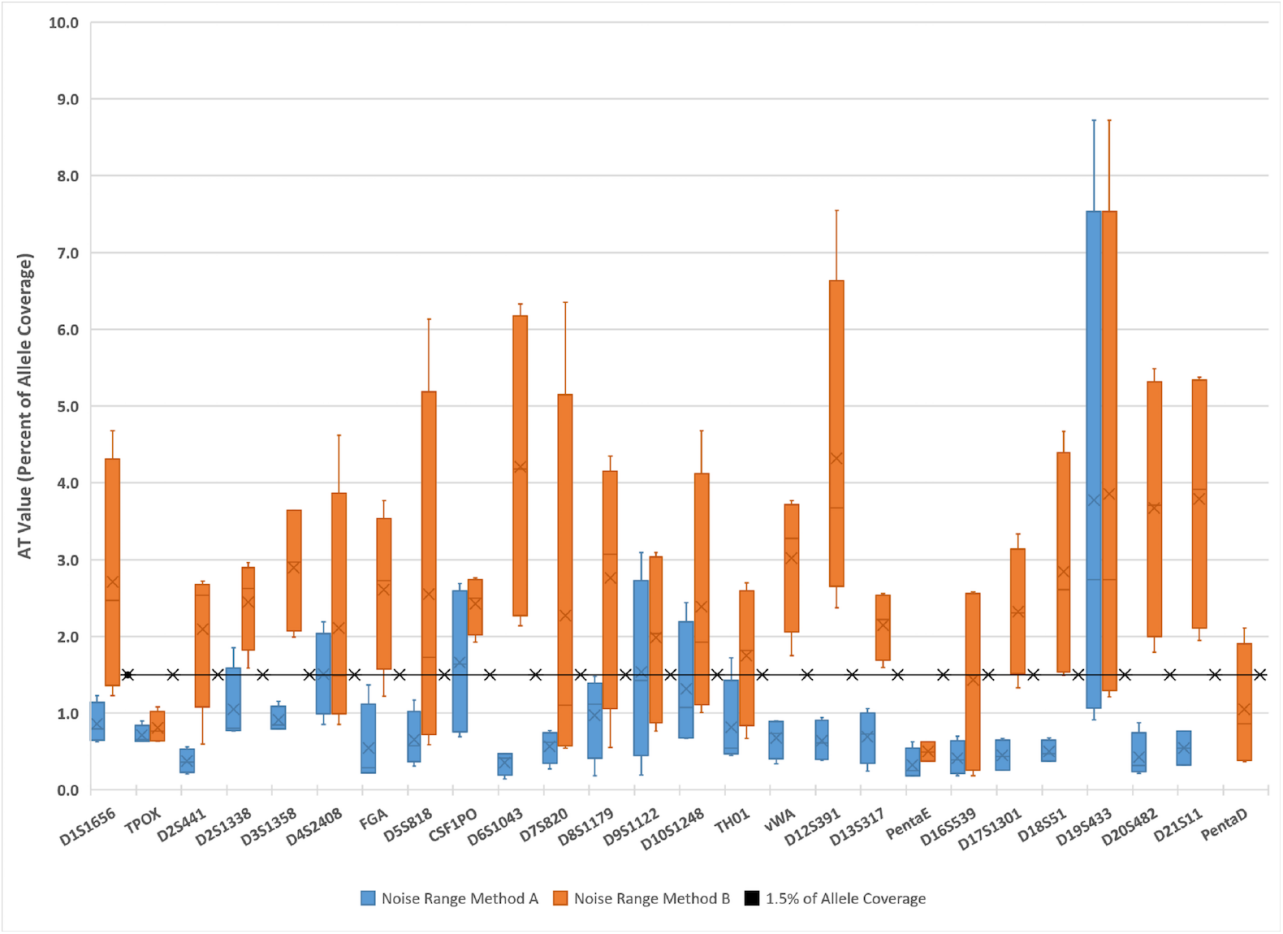
Comparison of analytical threshold (AT) values calculated from noise with ATs calculated as a percentage of allele coverage (signal). AT values by method A (blue boxes) are set at twice the range of observed per-locus noise intensities, where per-locus noise is defined as responses not attributable to alleles or molecular artifacts (stutter). AT values by method B (orange boxes) are set using the same formula, but where per-locus noise is defined as responses not attributable to alleles or N-1 molecular artifacts. The black line represents AT values set as a constant 1.5% of allele coverage. In all cases, AT values are converted to percentages of the average allele coverage on a per-locus basis.

Lacking a useful definition of noise, a common approach in PCR-MPS methods has been to define AT as a percentage of allele (i.e. signal) coverage intensity. This approach has been successfully implemented in prototype forensic PCR-MPS methods, but it has the disadvantage of not comprising a consideration of both signal and noise as recommended by SWGDAM. The default vendor recommendation used in Illumina ForenSeq^TM^ software is to set AT levels on a per-locus basis at 1.5% of the total locus read coverage. The total complement of reads assignable to a locus include authentic alleles (i.e. signal), molecular artifacts (i.e. systematic noise) and background noise, with most reads corresponding to allele sequences. Consequently, the ForenSeq method is dependent upon a combination of signal and noise intensity, with signal playing the largest role. A minimum total locus coverage of 650 reads is recommended, making the minimum possible AT value 10 reads when rounded up to the nearest whole read. In contrast, methods A and B depend only upon background noise intensities. When background noise is defined as the residual after removal of allele and all molecular artifact responses (method A) the AT levels tend to be slightly lower than the level calculated as 1.5% of locus coverage. When background noise is defined as the residual after removal of allele and N-1 molecular artifact responses (method B) the AT levels tend to be slightly higher than both the level calculated as 1.5% of locus coverage and by method A. The method B AT levels are more variable than method A AT levels (Fig 3).

**Fig 4:**
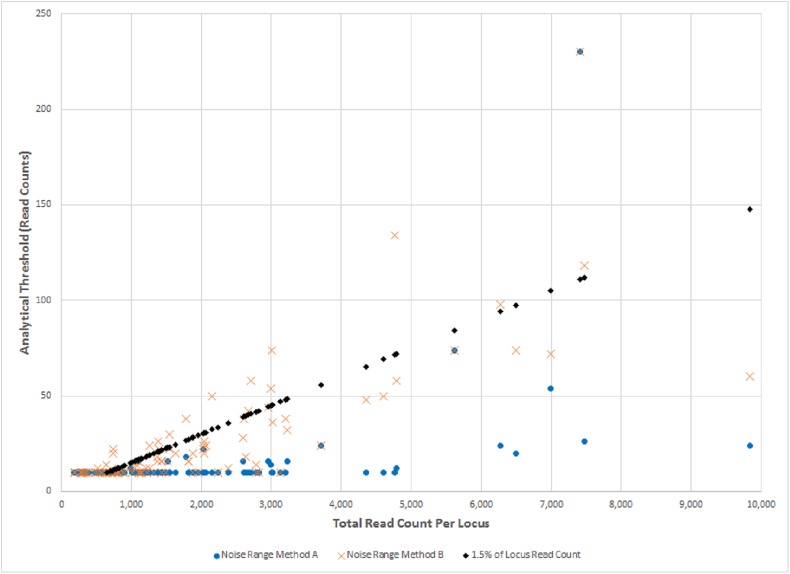
Effect of read coverage on analytical thresholds (AT) calculated by three different methods. Each data point represents a single instance of a sample-locus combination. AT values calculated as a fixed 1.5% percentage of total read coverage increase linearly with increasing read coverage (black diamonds) and are calculated for instances with a minimum of 650 total reads per Illumina ForenSeq protocol. This minimum corresponds to noise levels of 10 reads which for purposes of comparison has been used as the minimum AT value for all three methods. AT values based on background noise defined as the residual after removal of alleles and all stutter artifacts (Method A) are less sensitive to locus coverage (blue discs). An AT of 10 reads is generally sufficient for instances with coverages below 5,000 reads. AT values based on background noise defined as the residual after removal of alleles and N-1 stutter artifacts (Method B) trend upward with increasing locus coverage. Both Methods A and B are applied to instances with fewer than 650 reads.

AT values (expressed in read counts) set by the ForenSeq^TM^ method increase linearly with read coverage and values are locus and run-specific depending upon total read coverage for a locus and a run. AT values set by method B also increase proportionally to total read coverage. With method B, the AT values are driven by the maximum intensities of molecular artifacts, usually the N-2 or N+1 stutter artifacts. AT values set by method A are dependent upon background noise only and show less dependence on total read coverage (Fig 4). It may be possible to use a single AT value calculated by method A across a range of total coverage as is currently practiced in PCR-CE methods where AT is calculated from baseline fluorescence noise.

### Control of false positive rates

In forensic DNA analysis, the AT represents the threshold above which signal responses are interpreted as potentially authentic alleles. The AT also serves as a method detection limit (MDL) and, in this role, serves to set the false positive rate of the method. A false positive allele detection arises whenever a method artifact or background noise response is incorrectly interpreted as an allele response. Here, the three categories of types (authentic allele, molecular artifact, background noise) are explicitly identified by their characteristic intensity and nucleotide sequence features. The scaling factor in Eq 1 can be adjusted based on the level of risk desired against false positives arising from misinterpretation of background noise. This approach satisfies the SWGDAM recommendation to base AT values on considerations of signal and noise. The method is consistent with international guidelines for setting detection limits based on signal to noise ratios in analytical methods where scaling factors of 2 or 3 are typically recommended for peak-to-peak noise [27]. The method does not explicitly depend upon the distribution underlying background noise. Alternative methods for setting detection limits in PCR-CE methods generally assume an underlying Gaussian distribution for noise [12]. MPS background noise is not normally distributed, making these approaches inappropriate for PCR-MPS data. The underlying distribution is the subject of current research, and recent work by Vilsen et al. [28] indicates a more complex one-inflated, zero-truncated negative binomial distribution. The signal-to-noise method described here presents a straight-forward solution that is not dependent upon distribution parameters and is simple to implement in forensic laboratories.

SWGDAM recommendations also caution against setting AT values to obscure stutter artifacts. This recommendation was made in the context of PCR-CE fragment length analysis where stutter artifacts are usually limited to responses that size to N-1 and fit the expected intensity relative to an allele. The method described here satisfies this recommendation. The 26 loci of the four DNA samples contained a total of 190 sequence-defined alleles. Stutter artifacts were present for all alleles. Of these, a total of 175 stutter artifacts were observed to exhibit motif repeat numbers that place them in the CE-equivalent N-1 position. When calculation method A is applied on a per-locus basis, then 172 of 175 (98%) of these N-1 stutter artifacts have intensities above the obtained AT value. However, satisfying this recommendation is more complex in the context of PCR-MPS DNA sub-sequence analysis because the characteristic features of stutter are more complex. Sequence-defined stutter artifacts can be observed at a wider range of response intensities that can overlap with background noise and with a wide range of lengths. This phenomenon is particularly true for stutter artifacts at compound/complex loci where stutter artifacts can even be the same length as parent alleles. Therefore, while it is relatively straight-forward to set AT values such that N-1 stutter artifacts are above AT, it does not appear feasible to assure that any arbitrary level of complex stutter artifact is above AT. The method described here addresses this issue by explicitly identifying all stutter artifacts based on their nucleotide sequences relative to sequences identified as authentic alleles.

### Implementation

Various PCR-MPS workflows and sequencing platforms may exhibit workflow-specific levels of background noise. For example, workflows utilizing unidirectional reads (such as generated by ForenSeq^TM^ methods) may exhibit different type frequency spectrums than workflows utilizing bidirectional reads. Implementation of the method for either database or casework samples should therefore be workflow-specific.

## Conclusions

The method described here offers a solution to several challenges to defining background noise-based AT values as recommended by SWGDAM. First, it solves the problem of defining background noise in PCR-MPS methods. Second, the method provides an objective approach to setting AT values on an experiment, sample, or locus-specific basis. Third, the method provides an unambiguous definition for the DNA sub-sequence under analysis; and when common FSL are applied consistently to all read sub-sequences, then the sequence type count intensities for authentic alleles (i.e. signal) and background noise can be objectively linked for a given locus. Fourth, the AT method is independent of the level of multiplexing of marker panels or DNA samples in a sequencer run. This independence has practical implications for forensic analysis. Current PCR-MPS methods involve multiplexing of multiple marker panels. For example, the Illumina ForenSeq^TM^ DNA signature preparation kit used in this study multiplexed 27 autosomal STR markers with 32 other STR markers and 172 SNP markers. Any approach to noise measurement that depends upon counting reads that do not align to any target in the assay might require the examiner to analyze all panels for every inquiry regardless of whether the panels are of interest. This study focused specifically on autosomal STRs, and using the approach described, background noise could be measured without resorting to analyzing the other STR and SNP loci included in the ForenSeq^TM^ kit. In alignment-based sequence analysis reads that do not align to any target are directed to an “unaligned” file. The reads in this file can in one sense represent method noise. However, the number of unaligned reads can depend upon the level of multiplexing. If the focus of a forensic examination is limited to autosomal STR analysis, then the number of unaligned reads may differ depending upon whether, for example, ForenSeq^TM^ primer mixture A or B is used. By contrast, the method described here focuses on reads that can be identified as belonging to a specific primer pool at either the locus, panel, sample or experiment level. Thus, the reads included in the signal and noise analysis are limited to those relevant to the forensic analysis and AT calculations are unaffected by the level of multiplexing of loci not relevant to the analysis.

The use of the method described here allows forensic PCR-MPS methods to be brought into compliance with some of the standard expectations for forensic DNA analysis. These expectations include SWGDAM recommendations to base AT on considerations of signal and noise; as well as the common expectation in instrumental analysis to express method detection limits (i.e. AT) in the same units of measurement as used for signal and noise in the system. Whereas in PCR-CE methods, signal noise and AT are all measured in relative fluorescence units, the method described here allows these quantities to all be expressed in read count measurement units.

## Supporting information

S1 Table Flanking sequence landmarks (FSL) used to define loci under analysis. (DOCX)

S2 Table Sequences generated by the PCR-MPS method for 26 autosomal STR loci in sample number T9046. Sequences are categorized as allele, stutter or background noise and highlighted in green, yellow and gold respectively. Stutter sequences with CE size-equivalents of N-1 are highlighted in bold.
(XLSX)

S3 Table Sequences generated by the PCR-MPS method for 26 autosomal STR loci in sample number T23499. Sequences are categorized as allele, stutter or background noise and highlighted in green, yellow and gold respectively. Stutter sequences with CE size-equivalents of N-1 are highlighted in bold.
(XLSX)

S4 Table Sequences generated by the PCR-MPS method for autosomal 26 STR loci in sample number T28788. Sequences are categorized as allele, stutter or background noise and highlighted in green, yellow and gold respectively. Stutter sequences with CE size-equivalents of N-1 are highlighted in bold.
(XLSX)

S5 Table Sequences generated by the PCR-MPS method for 26 autosomal STR loci in sample number T36814. Sequences are categorized as allele, stutter or background noise and highlighted in green, yellow and gold respectively. Stutter sequences with CE size-equivalents of N-1 are highlighted in bold.
(XLSX)
