# Supplementary material for: A technique for setting analytical thresholds in massively parallel sequencing-based forensic DNA analysis
Source: PLoS One. 2017 May 18;12(5):e0178005. doi: 10.1371/journal.pone.0178005 (PMC5436856; doi:10.1371/journal.pone.0178005)
Supplement: S1 Table — (DOCX) [file pone.0178005.s001.docx]

Table S1. Flanking sequence landmarks (FSL) used to define loci under analysis

| **Locus** | **5'** | **Locus** | **3'** | **Chr.** | **Strand** |
| --- | --- | --- | --- | --- | --- |
| D1S1656 | CTGTRTGATG | STR | TTTAATTGTA | 1 | REV |
| TPOX | ACCCTCACTG | STR | GTTTGGGCAA | 2 | FWD |
| D2S441 | TATGAAAACT | STR | TATCATAACA | 2 | FWD |
| D2S1338 | GGCTTGGCCT | STR | CTCCTGCAAT | 2 | REV |
| D3S1358 | CTTGCATGTA | STR | TGAGACAGGG | 3 | FWD |
| FGA | TACAAGCTAG | STR | TTTCTTCCTT | 4 | REV |
| D4S2408 | AAAGCTATGC | STR | AATGGTTACT | 4 | FWD |
| D5S818 | AATATTTTGA | STR | GGTATAAATA | 5 | REV |
| CSF1PO | TAGATAGATT | STR | AGGAAGTACT | 5 | REV |
| D6S1043 | TTGATAGATC | STR | AGGATTTATT | 6 | REV |
| D7S820 | AYGAACTAAC | STR | GACAGATTGA | 7 | REV |
| D8S1179 | TACATTCGTA | STR | TTCCCCACAG | 8 | FWD |
| D9S1122 | TGTAGATAGG | STR | TATTAATAGG | 9 | FWD |
| D10S1248 | ATGAGTGAGW | STR | ATGAAGACAA | 10 | FWD |
| TH01 | CTCCATGGTG | STR | AGGGAAATAA | 11 | FWD |
| vWA | CTTGGATTGA | STR | WCTATCTGTC | 12 | REV |
| D12S391 | ATGCATAGGT | STR | GAGAGGGGAT | 12 | FWD |
| D13S317 | ACAAATACAT | STR | TTTTGGGCTG | 13 | FWD |
| PentaE | ACTCAGTCTC | STR | AAATTGTAAG | 15 | REV |
| D16S539 | SAGACAGGTG | STR | TCATTGAAAG | 16 | FWD |
| D17S1301 | CATATGTGTG | STR | CCATCATAGG | 17 | FWD |
| D18S51 | ACCTTGTCTC | STR | AAAGAGAGAG | 18 | FWD |
| D19S433 | GATTCTGTTG | STR | AGAGAGGAAG | 19 | REV |
| D20S482 | AACCAATAAG | STR | AGAGATTTAT | 20 | FWD |
| D21S11 | TGAATTGCCT | STR | TCGTCTATCT | 21 | FWD |
| PentaD | CCATCTCAAG | INDEL-STR | AAAAACGAAG | 21 | FWD |
